# Supplementary figures and images for: Investigation of lipolytic activity of the red king crab hepatopancreas homogenate by NMR spectroscopy
Source: PeerJ. 2022 Jan 3;10:e12742. doi: 10.7717/peerj.12742 (PMC8734460; doi:10.7717/peerj.12742)

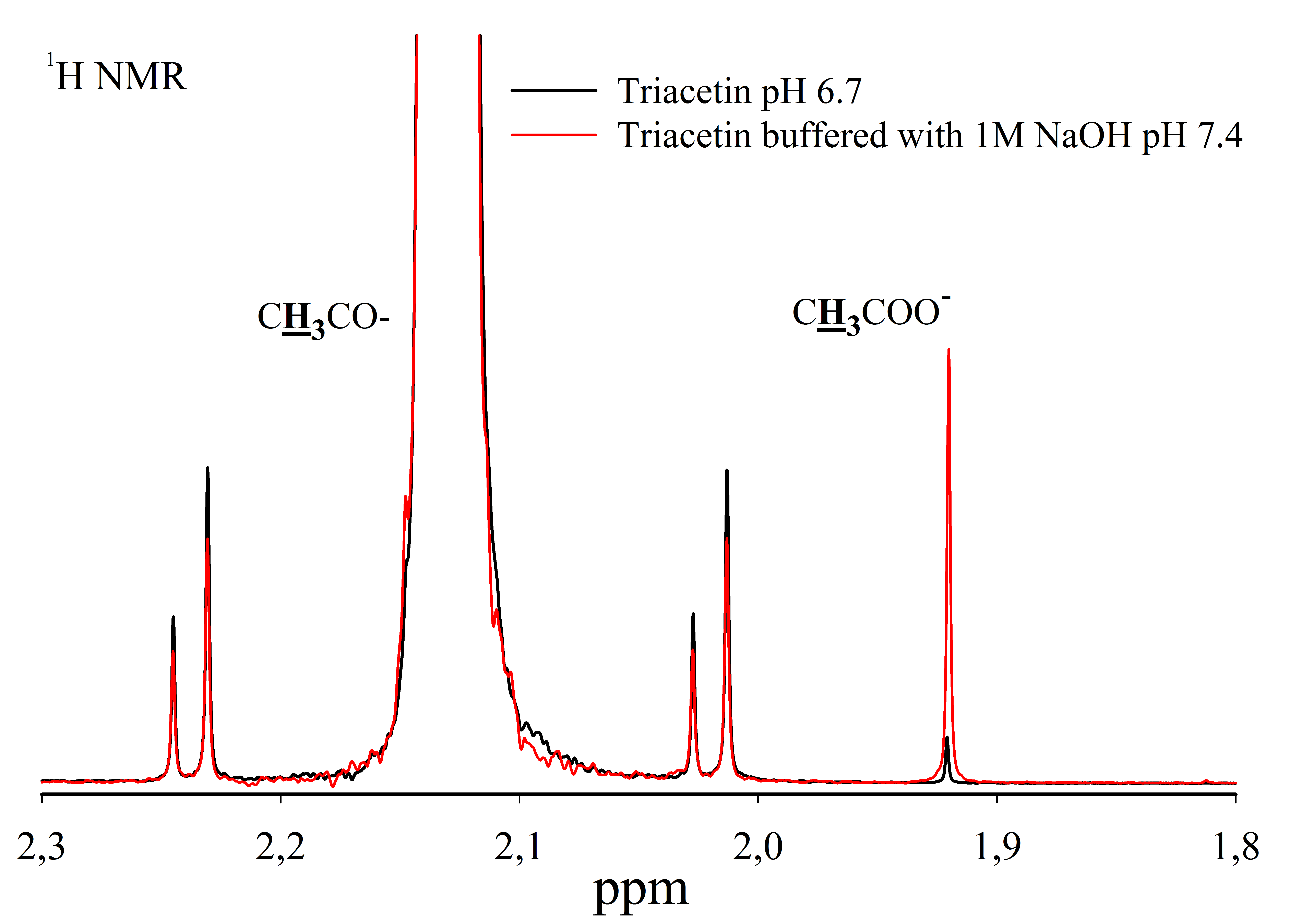

Supplement: Supplemental Information 1 — Protons that give signals are highlighted in bold and underlined. [file peerj-10-12742-s001.png]

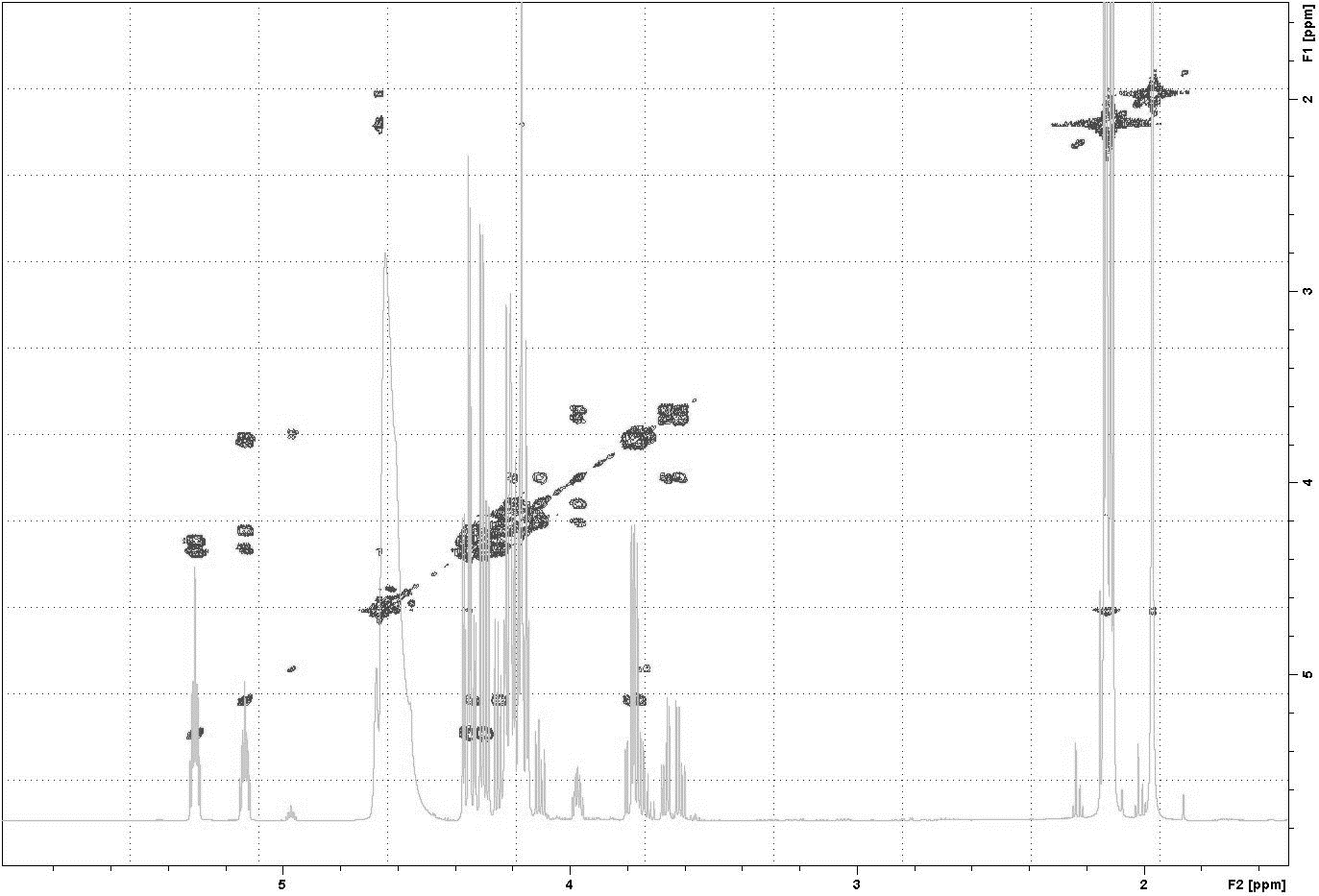

Supplement: Supplemental Information 2 [file peerj-10-12742-s002.png]

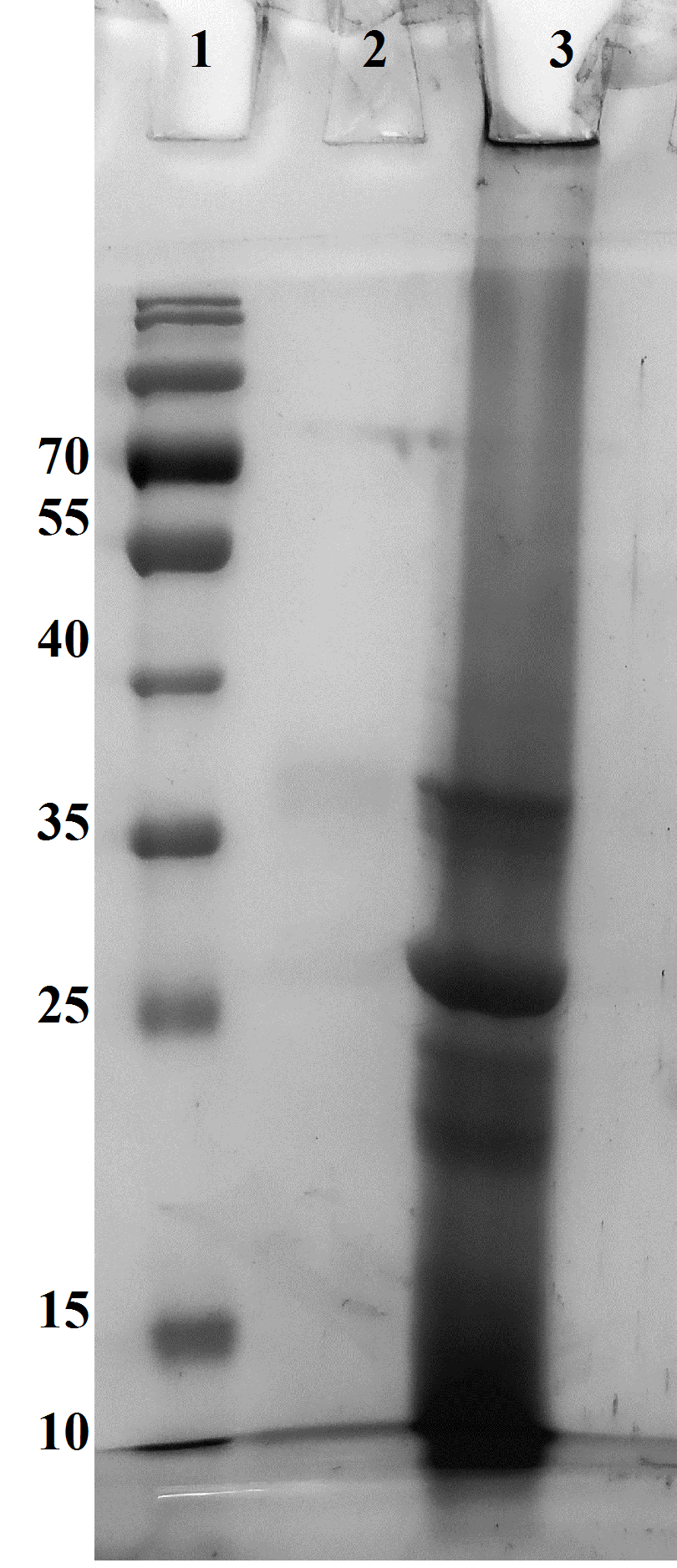

Supplement: Supplemental Information 3 — Lanes: (1) Molecular weight markers. (2) Soluble fraction of the NMR sample. (3) Precipitate in the NMR sample formed as a result of the reaction and dissolved in 15 µl of 50 mM phosphate buffer pH 7.2 with 8 M urea. [file peerj-10-12742-s003.png]

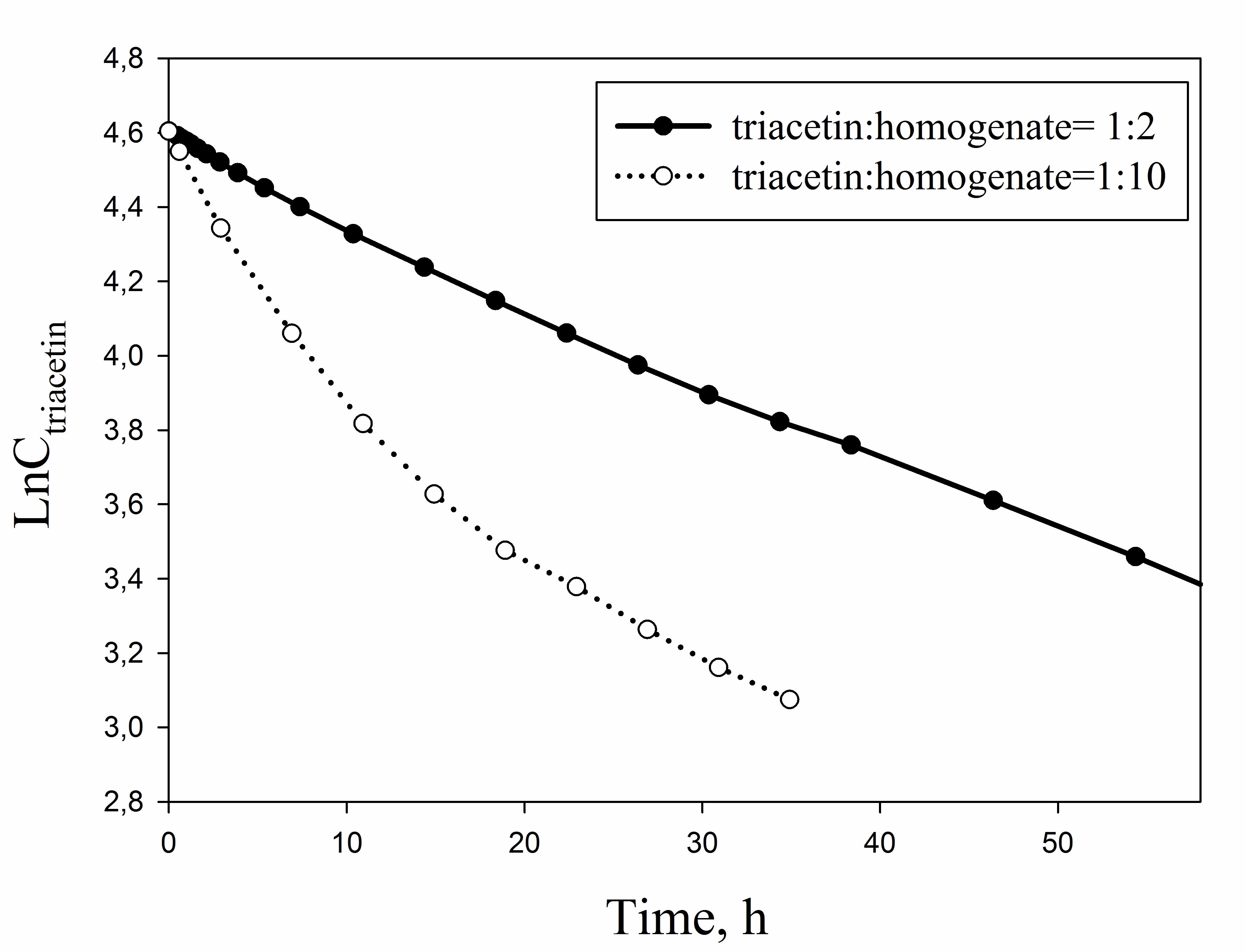

Supplement: Supplemental Information 4 [file peerj-10-12742-s004.png]

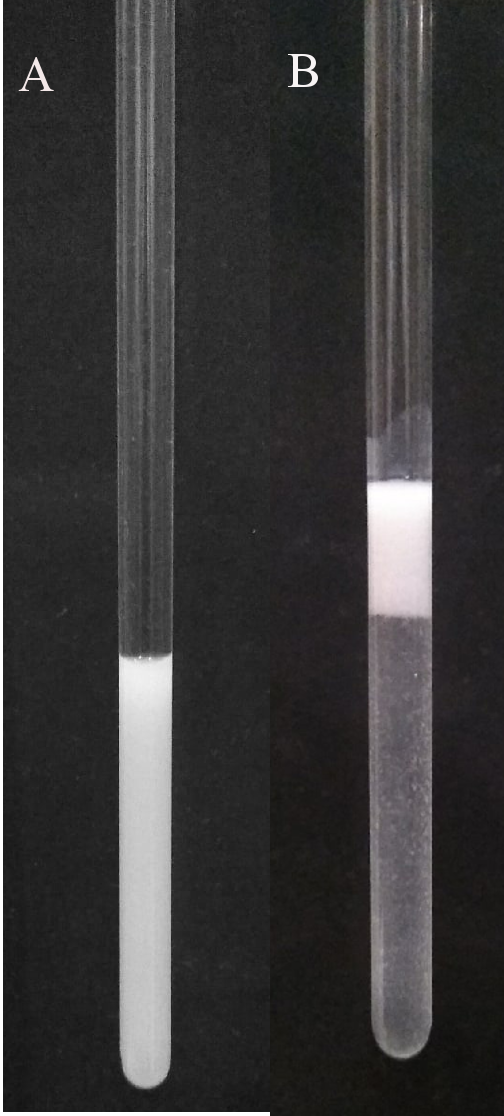

Supplement: Supplemental Information 5 — (A) An emulsion of caprylic/capric triglycerides before the addition of HPC homogenate. (B) The sample of caprylic/capric triglycerides after incubation with HPC homogenate for 35 h. [file peerj-10-12742-s005.png]

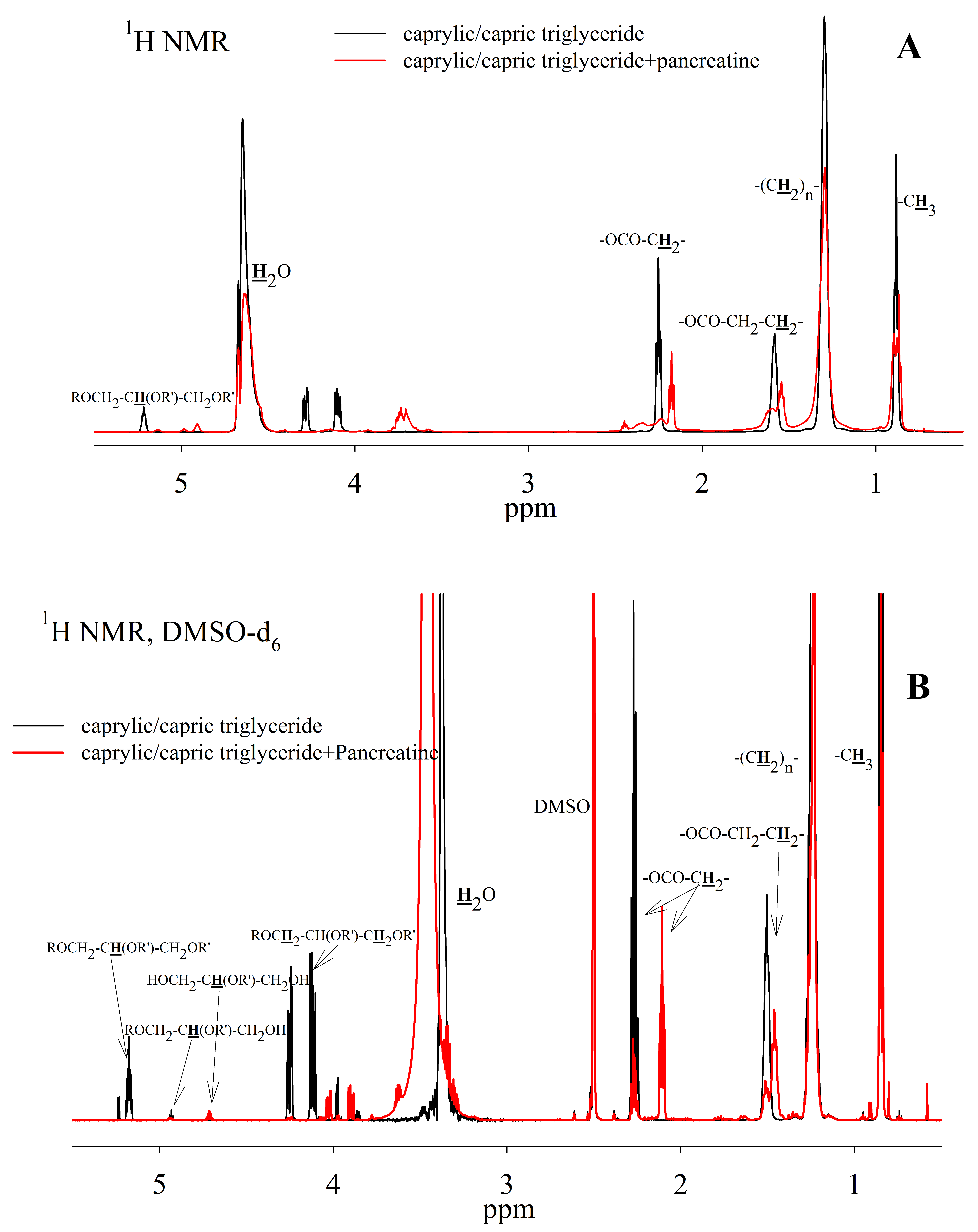

Supplement: Supplemental Information 6 — (A) The spectrum of the initial triacylglycerol and after hydrolysis. (B) The spectrum of reaction products contained in upper hydrophobic layer which was dissolved in DMSO-d6. The protons that give signals are highlighted in bold and underlined. [file peerj-10-12742-s006.png]
