# Supplementary material for: Investigation of lipolytic activity of the red king crab hepatopancreas homogenate by NMR spectroscopy
Source: PeerJ. 2022 Jan 3;10:e12742. doi: 10.7717/peerj.12742 (PMC8734460; doi:10.7717/peerj.12742)
Supplement: Supplemental Information 7 [file peerj-10-12742-s007.docx]

Table of chemical shifts.

| **№** | **Metabolite** | **Chemical shift and multiplicity [proton(s)]** | **NMR spectra** |
| --- | --- | --- | --- |
|  | TSP | 0.000 s [-Si(CH_3_)_3_] | 1D |
| 1 | TA | 2.1236 s [^2'^CH_3_];  2.1379 s [^2''^CH_3_];  4.3045 dd [^1b,3b^CH_2_];  4.3603 dd [^1a,3a^CH_2_];  5.3145 m [^2^CH] | 1D+2D(COSY) |
| 2 | 1,2-DA | 3.775 m [^3^CH_2_];  4.245 dd [^1b^CH_2_];  4.341 dd [^1a^CH_2_];  5.134 m [^2^CH] | 1D+2D(COSY) |
| 3 | 2-MA | 3.740 m [^1,3^CH_2_];  4.971 m [^2^CH] | 1D+2D(COSY) |
| 4 | 1-MA | 3.616 dd [^3b^CH_2_];  3.665 dd [^3a^CH_2_];  3.972 m [^2^CH];  4.105 dd [^1b^CH_2_];  4.205 dd [^1a^CH_2_] | 1D+2D(COSY) |
| 5 | Caprylic/capric triglyceride | 0.8828 t [^n'^CH_3_];  1.295 m [^4'-n'^CH_2_];  1.584 t [^3'^CH_2_];  2.252 t [^2'^CH_2_];  4.0995 dd [^1b,3b^CH_2_];  4.2842 dd [^1a,3a^CH_2_];  5.2159 m [^2^CH] | 1D+2D(COSY) |
| 6 | Caprylic/capric acids | 0.8828 t [^n'^CH_3_];  1.295 m [^4'-n'^CH_2_];  1.584 t [^3'^CH_2_];  2.1754 t [^2'^CH_2_] | 1D+2D(COSY) |
| Abbreviations: t - triplet; m – multiplet, s –singlet, d – doublet of doublets. TSP - Sodium trimethylsilyl propionate; TA –triacetin; 1,2-DA – 1,2-diacetin; 2-MA – 2-monoacetin; 1-MA – 1-monoacetin | | | |
