# Supplementary material for: Investigation of lipolytic activity of the red king crab hepatopancreas homogenate by NMR spectroscopy
Source: PeerJ. 2022 Jan 3;10:e12742. doi: 10.7717/peerj.12742 (PMC8734460; doi:10.7717/peerj.12742)
Supplement: Supplemental Information 8 [file peerj-10-12742-s008.doc]

**The calculation description (file *2021_GPK_nmr_p.xlsx)***

After the NMR spectra processing (folder ***mm210215***), the integral values of the components concentrations in the reaction mixture corresponding to the time of the spectrum registration were obtained.

The calculation of the components concentrations in the reaction mixture was carried out in the Microsoft Excel spreadsheet software program.

The first sheet of the file ***2021_GPK_nmr_p.xlsx*** (the sheet name is "tab") contains a table of assignments by chemical shifts of proton groups of components in ^1^H-NMR spectra.

The second sheet ("№№") contains the information about the data and time of experiments and a table with the numbers of ^1^H-NMR spectra and the corresponding time of their registration.

The third and fourth sheets ("I_C" and "I_C #", respectively) contain intermediate calculations and calculated component concentrations at fixed times (spectra registration times). The "I_C" sheet corresponds to the ratio of triacetin to HPC homogenate~1:2 (by volume) while "I_C #" sheet corresponds to the ratio of triacetin to HPC homogenate~1:10 (by volume).

Below is a description of the parameters given in the last two sheets "I_C" and "I_C #".

The lines 2 to 10 in the columns C and D describe which compounds are present in the reaction mixture and their amount. The cell (D, 10) shows the final concentration of the TSP standard in a sample placed in an NMR tube.

The lines 15 to 36 in the columns C - I comprise the integral intensities of the corresponding signals. The following abbreviations were used:

- **TA** – triacetin
- **1,2-DA** - 1,2-diacetin
- **2-MA** - 2-monoacetin
- **1-MA** - 1-monoacetin
- **Ac(A)** - total signals of the acetyl group in all glycerides
- **Ac** – free acetate
- **TSP** - 3-trimethylsilyl-[2,2,3,3-^2^H_4_] sodium propionate (TSP), internal reference

The columns M - S contain relative integral intensities relative to the TSP.

Based on the known TSP concentration, the integral concentrations of the mixture components (C*, μM / g, columns U - Z) were calculated.

To obtain the concentration values as close to real as possible, the correction factor between the concentrations of the formed di- and monoacetins from the formed free acetate was calculated (column AI). To calculate the real concentration of free acetate formed during the hydrolysis of triacetin, the concentration of the initially present acetate, which is not related to hydrolysis products, was subtracted from the integral concentration of free acetate (column AF). The correction factor (k(/)Ac) is the ratio of the real concentration of free acetate obtained (column AF) to the calculated concentration of acetate obtained from hydrolysis of TA (column AH), taking into account the integral concentrations of di- and monoacetins (columns V, W and X).

The concentrations of di- and monoacetins were calculated by multiplying their integral values by the correction factor (columns AC, AD and AE).

The real concentration of triacetin in the reaction mixture was determined by subtracting the concentrations of the formed di- and monoacetins from the initial concentration TA (column AB).

The use of this method for calculating the concentrations of components in the reaction mixture is due to the fact that in the process of enzymatic hydrolysis, a solution initially close to ideal loses its homogeneity. The molecules of triacetin, di- and monoacetins in micelles have lower mobility, which affects the decrease in their signals in the NMR spectra. At the same time, a small and charged acetate ion remains in the aqueous phase and partially retains its mobility, which makes it integral concentration closest to the real one. Therefore, all other concentrations were normalized to the acetate concentration.
